# Retraction: Skull Base Reconstruction Using a Multilayer Pericranial Flap After Cranioendoscopic Resection of Sinonasal Adenocarcinoma: A Single-Center Experience

**DOI:** 10.7759/cureus.r220

**Published:** 2026-02-20

**Authors:** José Alberto Fernandes, António Andrade, Carolina Santos Silva, Pedro Alberto Silva, Pedro Valente, Ricardo Vaz, Manuel Leal, Gil Coutinho

**Affiliations:** 1 Department of Otorhinolaryngology, Unidade Local de Saúde de São João, Porto, PRT; 2 Department of Neurosurgery, Unidade Local de Saúde de São João, Porto, PRT; 3 Department of Otorhinolaryngology, Hospital CUF Trindade, Porto, PRT

This article has been retracted by the Editor-in-Chief because it was submitted and published without appropriate departmental or institutional authorization. In addition, informed consent was not obtained for the publication of patient images, and departmental authorization was not granted for the use of intraoperative images. As a result, the article contents have been removed at the time of retraction.

